# Cross-sectional study to assess filarial infection among the never treated individuals in selected districts in India: a study protocol

**DOI:** 10.1136/bmjopen-2025-113797

**Published:** 2026-03-19

**Authors:** Raja J Dinesh, Adinarayanan Srividya, Muhammed Jabir, Manikandan Kishanthini, Vishal Dogra, Bhupendra Tripathi, Rinku Sharma, Tanu Jain, Manju Rahi

**Affiliations:** 1 ICMR - Vector Control Research Centre, Puducherry, India; 2 Gates Foundation, New Delhi, India; 3 National Centre For Vector Borne Diseases Control, New Delhi, India

**Keywords:** Mass Drug Administration, Tropical medicine, India, Public health, Neglected Diseases

## Abstract

**Abstract:**

**Introduction:**

With the global lymphatic filariasis (LF) elimination goal set to 2030, it is necessary to address challenges hindering the last-mile efforts. Never treated individuals are those who self-report that they have never taken the drugs for LF during any mass drug administration (MDA) rounds. Hence, it is necessary to identify these individuals and assess if they can be potential reservoirs of infection and understand the reasons for non-compliance.

**Methods and analysis:**

This mixed method study, proposed for a period of 2 years, will assess the filarial infection status of never treated individuals from four LF-endemic districts in India. A multi-stage cluster sampling design will be followed to select the health subcentres from one highly endemic block in each of the selected districts. A random sample of 2535 never treated individuals from each block will be assessed for filarial infection by a cross-sectional blood survey. Qualitative surveys, including in-depth interviews and focus group discussions, will be conducted to elicit the reasons for their non-compliance. The prevalence of filarial infection will be summarised as frequencies and percentages. Univariate and multivariate logistic regression analysis will be performed to find the factors associated with filarial infection. Exploring the various reasons, such as sociocultural, behavioural and programmatic drivers of non-participation, will enable the programme to design tailored communication and community engagement strategies to bring them under the umbrella of MDA and thereby support the ongoing LF elimination efforts.

**Ethics and dissemination:**

This study has been approved by the institutional ethics committee (IHEC 07-0824/N/F, dated 25 September 2024). After completion of the study, a workshop will be held with all stakeholders to disseminate the study findings.

**STRENGTHS AND LIMITATIONS OF THIS STUDY**

- A major strength of this protocol is its mixed-method design, as it integrates both epidemiological and social science approaches to understand various factors influencing their systematic non-participation in mass drug administration.
- A large sample size with sufficient power will be used to estimate filarial infection prevalence among the never treated
- Awareness of lymphatic filariasis will be imparted to the never treated individuals.
- Identification of never treated individuals retrospectively from the previous 5 years, which could be influenced by potential recall bias.
- Capturing the out-migrant population is yet another challenge.

## Introduction

 The Global Programme for Elimination of Lymphatic Filariasis (GPELF), initiated in 2000,^1^ targeted to eliminate lymphatic filariasis (LF) as a public health problem globally by 2020.^2^ With the identification of new endemic areas using the new confirmatory mapping tool^3^ and several areas continuing preventive chemotherapy, the global target for elimination was shifted to 2030.^4 5^

The GPELF strategy consists of two core pillars: (1) mass drug administration (MDA) and (2) morbidity management and disability prevention (MMDP). While the MDA advocates yearly administration of antifilarial drugs to all eligible individuals in endemic areas to achieve interruption of transmission, the MMDP recommends a minimum care package to alleviate suffering due to chronic disease.^6^ For MDA, the WHO recommends the use of different drug regimens: (1) two drugs DA–6 mg/kg of body weight diethylcarbamazine (DEC) with 400 mg albendazole or IA–150 µg/kg of body weight ivermectin with 400 mg albendazole in areas co-endemic for onchocerciasis for at least 5 years, (2) single-drug (400 mg albendazole, preferably twice per year in areas co-endemic for *Loa loa*) for at least 10 years and (3) triple-drug (IDA–200 µg/kg ivermectin with 6 mg/kg DEC and 400 mg albendazole) for at least 2–3 years.^7 8^ The WHO recommends a minimum threshold of ≥65% as effective coverage during each MDA round to reduce microfilaria (Mf) prevalence below 1%.^8^

India, with 348 endemic districts (across 20 states and union territories),^5^ launched its national campaign to eliminate LF in 2004.^9^ Initially, the National Health Policy (2002) targeted LF elimination by 2015, which was later aligned with the global LF elimination targets.^9 10^ The IDA-MDA strategy was introduced as a part of an accelerated plan to eliminate LF in 2018,^11^ and recently an enhanced five-pronged strategy was launched to tackle LF.^12^ This strategy included mission-mode MDAs aligning with the biannual national deworming day covering endemic areas in phases, strengthening MMDP services by involving local medical colleges, multisectoral approaches towards integrated vector control, high-level advocacy with allied departments/ministries, use of available digital platforms for LF and exploration of newer diagnostic tools.^13^

As of 2024, 141 districts stopped MDA after passing the first transmission assessment survey (TAS), and another 43 are at various stages of Pre-TAS/TAS or the IDA Impact Survey.^5 13 14^ Despite more than two decades of programmatic efforts, 159 districts (across 13 states) are still implementing MDA, and five districts failed to qualify for Pre-TAS.^9 13^ Although successful to some extent, the national programme had faced several implementation challenges.^15^ One among the several challenges faced in the community is the issue of those individuals who missed MDA^15^ or the ‘never treated’, that is, those individuals who never received even a single dose to date.^12^ Never treated is a term that refers to individuals who self-report to have never been treated for LF during any MDA round and impacts global and national efforts towards LF elimination.^8 16 17^ These individuals may have missed MDA for reasons that may be either intentional (refusal because of fear of side effects or misinformation, mistrust in government programme/drug, low perceived risk of disease) or unintentional (not available at the time of MDA, unaware of MDA, ineligibility due to repeated pregnancies/lactation).^17^ A few studies have reported that the filarial infection rates are significantly higher among those who never participated in MDA or took LF drugs in only one round of MDA.^18^,^20^ Brady *et al* reported that although there is evidence to support that the people never taking drugs clear infection at a much slower rate, little is known about their impact on ongoing transmission and stressed the need for more in-depth studies to understand the problem of never being treated.^17^ The implication of never treated individuals on LF transmission was also one of the high-priority areas identified by LF experts during the WHO Regional Technical Advisory Group meeting held in Nepal in June 2024.^21^

In India, several studies have identified individuals who are frequently missed during MDA, including migratory populations, communities living in remote areas, nomadic and marginalised groups, and individuals in urban and slum areas.^22^,^24^ The proportion never treated, estimated by studies in India, ranges between 16.5% and 41.5%.^25^,^27^ These individuals may serve as hidden reservoirs of infection and aid in continued transmission.^17 28^ However, there is a paucity of evidence on the size, demographic and socioeconomic characteristics, infection status and reasons for their non-participation.

Therefore, this study protocol is designed to assess the role of never treated individuals in LF transmission in four highly endemic districts in India with the following objectives:

1. to estimate the prevalence of filarial infection (CFA and Mf) among the never treated individuals and
2. to determine the various factors, such as gender perspectives, occupation, religious beliefs, physical ailments and barriers impeding their access and participation in MDA.

The study adopts an integrative design, combining both epidemiological and social science approaches to assess the infection status of never treated individuals and the factors associated with their systematic non-adherence. It will be implemented by the Indian Council of Medical Research–Vector Control Research Centre (ICMR-VCRC), a WHO collaborative centre for research and training on LF and integrated vector management, in partnership with the National Center for Vector Borne Diseases Control (NCVBDC), New Delhi, and the respective state programmes with financial support from the Bill and Melinda Gates Foundation.

## Methods

### Study settings

**Figure 1:**
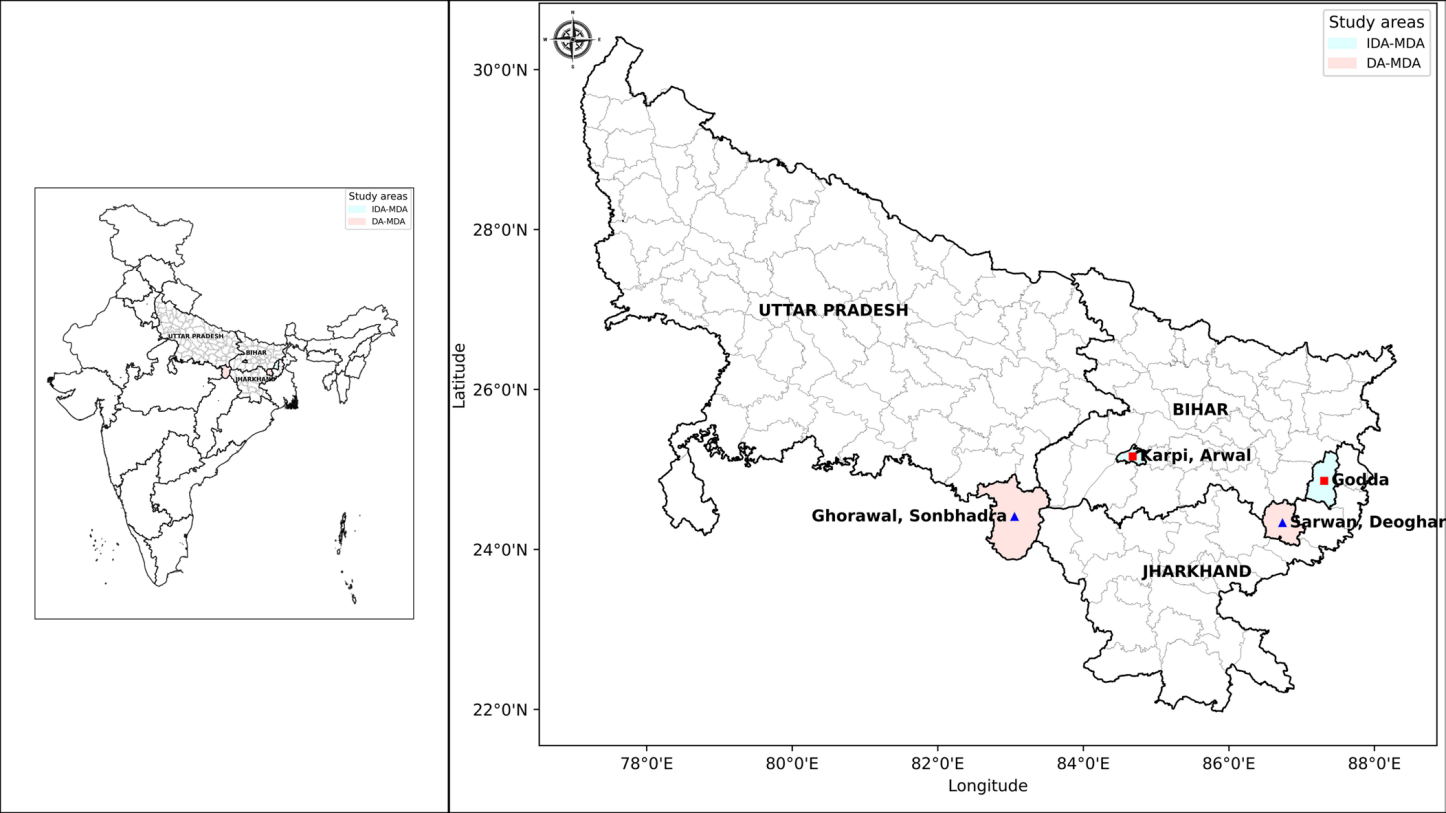
Map showing the study areas (district, state): Arwal in Bihar, Sonbhadra in Uttar Pradesh and Godda and Deoghar in Jharkhand (Map created using an India map available from http://www.surveyofindia.gov.in/ and Python V.3.12). DA, diethylcarbamazine and albendazole; IDA, ivermectin, diethylcarbamazine and albendazole; MDA, mass drug administration.

This cross-sectional study will be conducted in four endemic districts selected in consultation with the NCVBDC, where two of each are currently under DA-MDA and IDA-MDA: Arwal (IDA) in Bihar, Sonbhadra (DA) in Uttar Pradesh, and Godda (IDA) and Deoghar (DA) in Jharkhand (figure 1). The districts were selected in consultation with the national programme.

**Figure 2:**
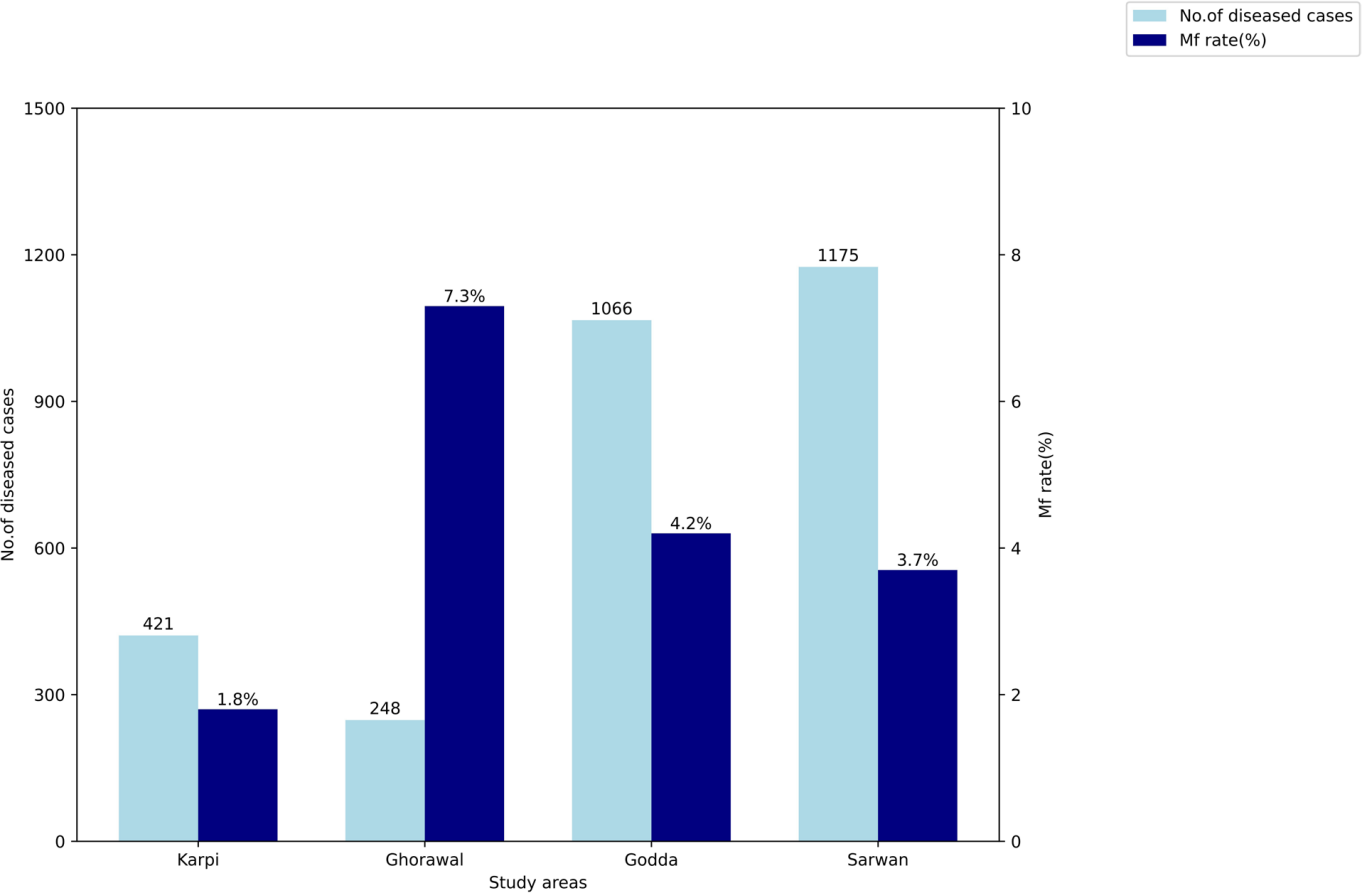
Microfilaraemia (Mf) rate and number of chronic lymphatic filariasis cases in the selected blocks.

One block each from these districts with a high microfilaremia (Mf) rate (based on night blood surveys conducted in 2024) was selected as the study site as shown in table 1, and these also recorded a high number of diseased cases as seen in figure 2.

**Table 1:** Details of the four study sites

District and state	Blocks	Type of MDA area	Total number of MDA (DA/IDA) rounds as of 2025	Population	Mf rate (%) (2024)*
Arwal, Bihar	Karpi	IDA (since 2019)†	13	199 466	1.8%
Godda, Jharkhand	Godda	IDA (since 2023)†	18	294 796	7.3%
Sonbhadra, Uttar Pradesh	Ghorawal	DA (since 2004)	16	282 998	4.2%
Deoghar, Jharkhand	Sarwan	DA (since 2004)	18	225 351	3.7%

* These figures were obtained from the respective state National Center for Vector Borne Diseases Control.

† These IDA areas were earlier under DA-MDA since 2004.

DA, diethylcarbamazine, albendazole; IDA, ivermectin, diethylcarbamazine, albendazole; MDA, mass drug administration; Mf, microfilaria.

### Operational definition of never treated

Discussions were held with the stakeholders, like the national programme, LF experts, the WHO and implementers in the field. As a few other national programmes also adopt the MDA strategy (like the programme for soil-transmitted helminths), and to minimise recall bias, the stakeholders came to a consensus to consider only those who did not participate in the previous five consecutive rounds of MDA (DA or IDA). Accordingly, an operational definition was conceived as: ‘Never treated are those individuals who have missed participation in the previous five consecutive MDA rounds in the area’.

### Study population

All consenting individuals identified from the MDA registers or through Accredited Social Health Activists (ASHAs)/Auxiliary Nurse Midwives (ANMs) or personal interviews during house visits who self-report to have not participated in the previous five consecutive MDA rounds will be the study population.

Inclusion criteria: Any individual satisfying the operational definition for never treated and willing to participate in the study will be included. During enumeration, if an entire family is never treated, all members of the family above the age of 2 years will be screened for filarial infection.

Exclusion criteria: Children aged less than 2 years and those individuals found to be seriously ill or bedridden at the time of the survey will be excluded.

### Survey methodology

#### Sample size for quantitative surveys

Blood survey for filarial infection: To assess the filarial infection prevalence among the never treated, assuming a Mf prevalence of 1%, 0.5% precision, a design effect of 1.5 and 10% non-response, a minimum sample size of 2535 was estimated per block. Thus, a total of 10 140 never treated individuals will be screened for filarial infection from four blocks.

#### Sample size for qualitative surveys

KAP surveys: To assess knowledge, attitudes and practices (KAP) related to LF and participation in MDA, assuming a 33% awareness of LF among the individuals,^29^ 3% precision, a design effect of 1.5 and a non-response rate of 10%, 1575 never treated individuals will be interviewed from each block. This sample will be proportionately allocated among the enumerated never treated individuals in the 30 selected clusters.

A stratified purposive sampling approach will be applied to select never treated participants for qualitative interviews. Participants will be stratified by type of non-participation in MDA–intentional (those who were aware of the programme but chose not to participate) and unintentional (those who missed the MDA due to factors such as lack of access or logistical challenges).

20 in-depth interviews (IDIs) will be conducted with the never treated individuals, including 10 intentional and 10 unintentional non-participants in each block. Within each stratum, participants will be purposefully selected to ensure diversity in gender, age, socioeconomic status and geographic distribution, including representation from both remote and centrally located areas within the block. Although the initial sample size is set at twenty interviews per block, the final number may be adjusted based on data saturation. Further, to understand the supply-side related perspectives, five interviews each will be conducted with health system staff from district NCVBDC and non-health sector representatives (teachers, self-help groups, non-governmental organisations and panchayat members), respectively. Two focus group discussions (FGDs) will be conducted with community drug administrators (CDAs) per block.

### Sampling methods

A multistage sampling method will be adopted. In stage I, from each of the selected endemic districts, one high endemic block that reported high Mf infection rates in the recent annual night blood surveys was selected. In stage II, 30 health subcentres will be selected using the probability proportionate to estimated size method. Health subcentres are the first level of contact between the community and the primary healthcare system in the country that cater to a population of approximately 5000, constituting 3–10 villages. If any block has fewer than thirty health subcentres, all of them will be chosen. In stage III, from each selected health subcentre, the never treated population will be enumerated.

Enumeration of never treated individuals: Village-wise MDA registers available with the ASHAs and ANMs will be accessed and scrutinised to identify never treated individuals in the past five rounds. ASHAs and ANMs are village-level healthcare workers who act as a link between the community and the primary healthcare system in the country, catering to a population of approximately 1000. They are local residents of the village and are well known to the people in the community, as they carry out regular household visits as a part of their responsibilities. They usually maintain a family register containing sociodemographic and other healthcare-related details of all individuals in the households in their allotted area. Further, they are also well trained to be drug administrators during the MDA rounds and maintain records in the form of MDA registers, which contain sociodemographic details of beneficiaries and defaulters. In case the information on never treated is not available in the registers, the ASHAs and ANMs will be probed in-depth to identify these individuals, as they are usually aware of individuals/families who never consumed or migrated at the time of MDA rounds. If MDA registers are not available in any area or if the ASHAs/ANMs have no such information, households will be systematically selected, and the individuals will be probed for non-participation during MDA till the required sample size for that village is achieved. Information will also be supplemented with the data from other sources like WHO monitoring teams, non-governmental organisations and coverage evaluation surveys, if available. Individuals identified from the registers or through the healthcare workers will be randomly cross-verified by visiting the households to ascertain their non-participation in the previous MDA rounds.

Sampling procedure: On completion of enumeration of never treated individuals in 30 health subcentres, the total sample size will be proportionately allocated based on the enumerated individuals in each health subcentre. Subsequently, the sample size allocated to each health subcentres will be further proportionately allocated to the total enumerated in each of the villages under the health subcentre. Adequate efforts will be taken to cover individuals from all age classes. In villages with high refusal rates or non-availability of enumerated individuals, the adjacent houses will be visited and probed about their participation in MDA. Any never treated individual identified in this process will be included to achieve the target sample size for the village.

**Figure 3:**
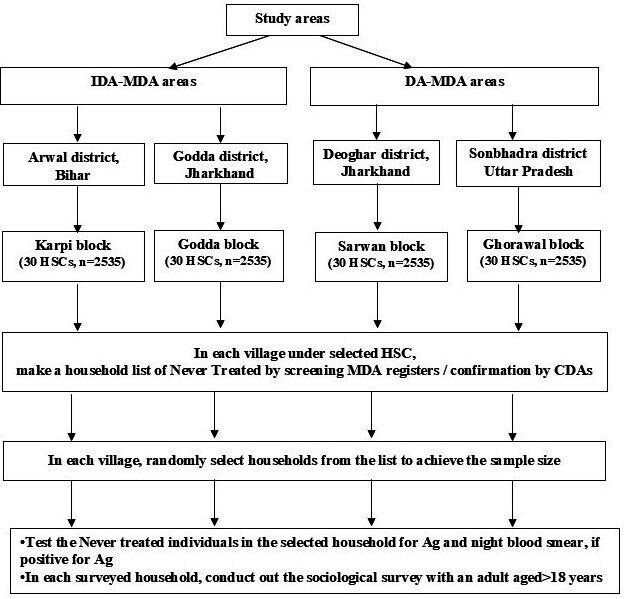
Flow chart showing the sampling plan of the study. Ag, filarial antigen; CDA, community drug administrator; DA, diethylcarbamazine and albendazole; HSC, health subcentre; IDA, ivermectin, diethylcarbamazine and albendazole; MDA, mass drug administration.

The sampling method in the four study districts is shown in figure 3.

### Survey tools and procedures

Using the enumerated list of never treated individuals in each village of the selected health subcentres, household visits will be done to enrol the participants. The individuals will be explained about the purpose of the study and the procedures involved. Only those consenting individuals will be assessed for circulating filarial antigen (CFA) using the STANDARD Q Filariasis Antigen Test (QFAT) kits^8^ with comparable sensitivity and specificity to filariasis test strips^30 31^ and those positive for CFA will be screened further for Mf by night blood smear the same night.

- CFA testing: About 20 µL of capillary blood collected by a finger prick using a micropipette will be placed on the test pad, and two drops of chase buffer will be added to the well. The test results will be read exactly at the end of 20 min as ‘positive’ or ‘negative’ as per manufacturer instructions.^8^ Any invalid test will be repeated once.
- Night blood smears**:** Individuals positive on QFAT will undergo Mf testing between 22:00 and 24.00 hours. Approximately 60 µL of capillary blood will be collected, and three parallel lines of 20 µL each (smears) will be made on the slide. Slides will be air-dried, packed and later stained following standard procedures by the WHO.^8^ The smears will be microscopically examined independently by two trained technical staff at ICMR-VCRC. To ensure quality, all positive slides and 10% of the negative slides will be cross-examined by a senior technical staff.

**Figure 4:**
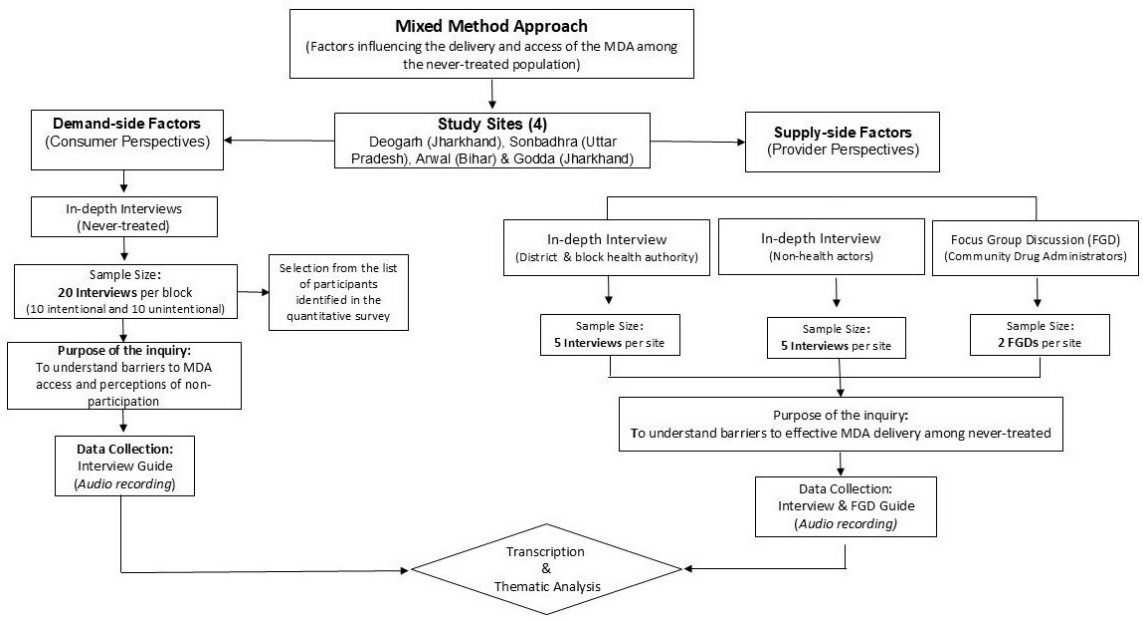
Flow chart for sociological survey. FGD, focus group discussion; MDA, mass drug administration.

The qualitative component of this study will employ IDIs and FGDs to understand the prevailing perceptions and various factors influencing MDA non-participation (figure 4).

- KAP Surveys: Face-to-face interviews with an adult household member will be conducted using a structured questionnaire. The survey will capture details on socio-demography and knowledge of LF, including the modes of transmission, symptoms, knowledge of preventive measures and sources of information. Perceived severity of disease, perceived infection risk, beliefs regarding the safety of MDA drugs and willingness to participate in future MDA rounds will be used to assess their attitude. Practice-related components will capture details on previous participation in MDA, including receipt and consumption of drugs, reasons for non-receipt or non-compliance, and participation of other household members. The survey will also document usage of personal protective measures and self-reported morbidity such as lymphedema or hydrocele.
- IDIs: On the demand side, interviews (using a semistructured interview guide) will specifically target never treated individuals identified and tested through quantitative surveys. Face-to-face interviews will be conducted with participants, lasting approximately 1 hour each. Tailored interview guides will be developed to ensure systematic exploration of their knowledge and perception of LF, existing community norms, perceived risk and preventive practices, awareness and past experiences with MDA. Further, the barriers and enablers related to MDA, including socio-cultural factors, concerns about drug safety, influence of family and peers, migration or work-related constraints, access and timing of drug distribution and interactions with drug administrators, will be probed. Additionally, their willingness to participate in future MDA rounds and suggestions to improve programme reach and acceptability will also be captured. On the supply side, the interviews will be conducted with key informants from health systems such as district health officers and vector-borne disease control officers who play key roles in planning, coordination and on-ground drug administration activities. These interviews will gather information on community response to the MDA programme, identification and characteristics of never-treated populations, common reasons for non-compliance, existing tracking and follow-up mechanisms, and previous interventions targeting never-treated populations. In addition, stakeholders representing non-health sectors such as school teachers, members from self-help groups, representatives of local institutions and non-governmental organisations will be interviewed. These actors are often instrumental in community engagement and can provide valuable perspectives on outreach efforts and the broader socio-cultural factors that influence MDA participation. Separate interview guides will be prepared to gather information on their involvement in MDA activities, nature of engagement, perceptions on community participation, perceived reasons for non-participation, experience of engaging with never-treated individuals, effectiveness of existing community engagement activities, operational challenges and suggested strategies for improving inter-sectoral involvement in MDA administration.
- FGD: The FGDs with community drug administrators such as ASHAs and ANMs will be facilitated by a trained moderator from the research team, with assistance from supportive staff for audio recording and note taking. An FGD guide will be used to guide the discussions and will focus on field-level barriers in reaching out to never treated individuals and factors that prevent community participation in MDA rounds. The discussions will take place in accessible community spaces (eg, community hall or anganwadi) and will be conducted in local languages (primarily Hindi). All sessions will be digitally recorded using audio recorders.
- All survey tools, including questionnaires and interview guides, will be pilot tested before deployment.

### Training of project staff

Project staff for the fieldwork will be locally hired to ensure familiarity with the local language and cultural settings. They will be adequately trained by qualified technicians from the institute prior to the field surveys. A trained sociologist will train the staff in conducting the qualitative surveys.

### Data management and quality control

Data collection using REDCap: Survey data will be collected electronically using tablets through the REDCap mobile application. A custom REDCap mobile application was developed with three forms: (1) personal details to capture demographic and individual-level information, (2) filarial infection status based on QFAT and (3) a KAP survey form to collect information on social, economic and behavioural aspects from the participants.

Field staff will be adequately trained on the use of the application and the process of filling the formats. Data will be entered offline in the field and stored securely on the devices. After completing the surveys each day, the data will be synchronised to a centralised REDCap server at the institute by connecting to the internet in the field, enabling real-time access for monitoring.

### Quality control

The principal investigator and coinvestigators will closely monitor data collection on a daily basis and check for any errors in the data throughout the study. Any inconsistencies will be communicated to the field teams for correction immediately, after which the corrected data will be re-synchronised. Storage and access will be managed according to the institutional data protection protocols, and local copies will be deleted from the devices to ensure confidentiality and prevent data overwriting.

### Data analysis

Using the data collected in the survey, the CFA and Mf positivity of never treated individuals will be summarised as frequencies and percentages with 95% CIs. Univariate analysis will be performed to find the association of infection status (Mf/CFA positivity) with various variables such as age, gender, education, occupation, socioeconomic status and duration of residency. The inclusion of variables in the multivariate analysis will be based on the initial p-values from the univariate analysis. Multivariate logistic regression will be performed to identify the factors associated with infection in the never-treated population.

Qualitative data from IDIs and FGDs will be digitally recorded and transcribed to English and thematically analysed using the Atlas.ti V.23.2.1 software.^32^ A grounded theory approach will be used for the development of the coding framework. To strengthen the findings, triangulation will be employed across data sources (IDIs and FGDs) to identify both converging and diverging patterns. The study findings will be organised under key themes and categories derived from the analysis. Direct quotations from participants and network diagrams will be used to depict key characteristics and barriers among the never treated individuals.

### Study status

The study is approved for a period of 2 years (from November 2024 to November 2026). Administrative approvals and permission were obtained by June 2025, following which the study was initiated in one of the sites in July 2025 and is currently ongoing.

### Outcome measures

In this study, we propose to estimate certain outcome measures in terms of indicators which will be of use for the programme to understand the magnitude of the problem of never treated individuals in an endemic area, the probable risk of LF transmission in terms of their infectious status, the reasons for their systematic non-compliance, and also the barriers in terms of supply chain and lack of manpower during the implementation phase. The outcome measures and their indicators we propose to estimate from this study area are given in table 2.

**Table 2:** Outcome measures from this study on never treated in an endemic area

Outcome	Indicators
Proportion of never-treated population	Percentage of never treated identified through screening of MDA registers
Prevalence of filarial infection	Prevalence of circulating filarial antigenemia among tested participants Proportion of participants testing positive for microfilaremia by night blood smears
Knowledge and awareness of lymphatic filariasis and mass drug administration (MDA)	Proportion of participants aware of lymphatic filariasis, its transmission mode, symptoms and preventive measures Proportion of participants aware of the MDA programme and its purpose
Reasons for non-participation (demand-side)	Self-reported barriers among never treated individuals (eg, lack of awareness, fear of side effects, mistrust, cultural belief, logistical constraints) Categorisation of non-participation into intentional versus unintentional
Perception of MDA implementation (supply side)	Qualitative insight on challenges in programme delivery, including workforce adequacy, supply chain gaps, coverage levels, community involvement, access, etc Perception of strategies used to reach hard-to-reach/never treated populations

## Discussion

The GPELF, since 2000, has made tremendous progress, with 33 out of 71 endemic countries having stopped MDA.^5 8 14^ However, many countries have raised concern regarding the risk of transmission associated with individuals who have never participated in any of the MDA rounds.^21^ As several other endemic nations will stop MDA and enter the post-MDA surveillance phase in the near future,^5 8^ it is pertinent to take concrete efforts to identify and treat individuals who have never consumed the MDA drugs either intentionally or unintentionally. Several research and modelling studies have demonstrated that these individuals can remain potential reservoirs for ongoing transmission, thereby hindering the global efforts towards LF elimination.^17 19 28 33 34^ Various terminologies such as ‘systematic non-compliance’, ‘persistent non-compliance’, ‘systematic non-adherence’, ‘systematic non-uptake’ and several others have been used so far to describe these individuals in published literature.^1933 35^,^38^ However, the term ‘never treatment’ was proposed and accepted by members of the neglected tropical diseases community in 2021^17^ and the same has been introduced in the recently released monitoring and evaluation guidelines by the WHO.^8^

**Figure 5:**
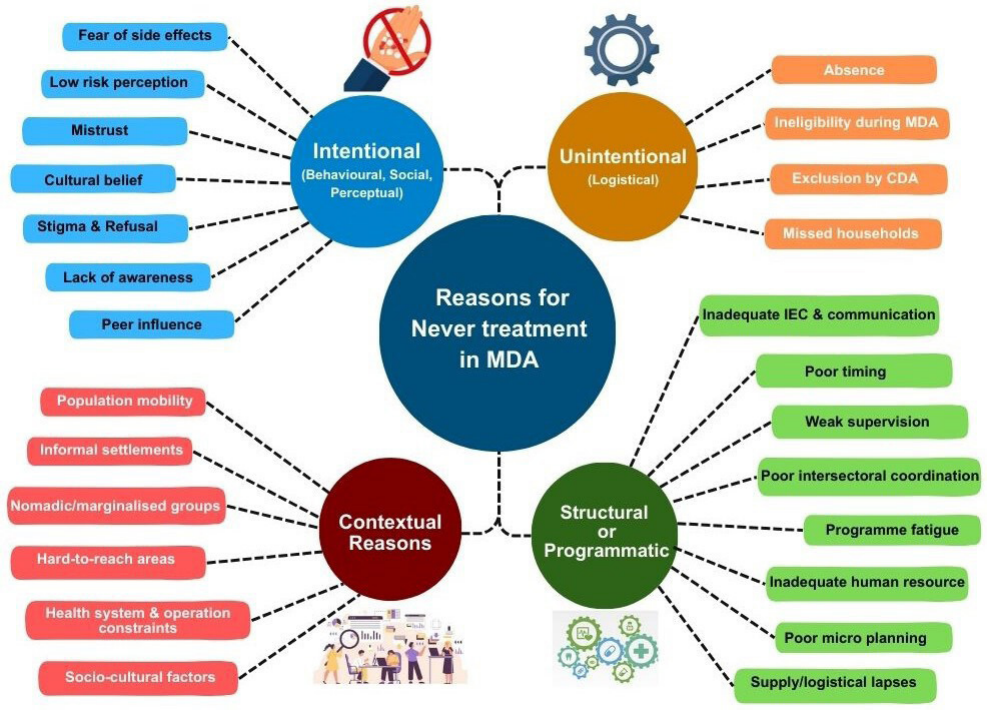
Reasons for never treatment based on literature review. CDA, community drug administrator; IEC, information, education and communication; MDA, mass drug administration.

Although previous studies have documented the reasons for community non-participation during MDA, based on coverage evaluation surveys following a single round of recently completed MDA.^22 26 37^ A coverage evaluation survey conducted among 328 individuals aged 18–75 years from an evaluation unit in Nagpur, India, reported that 33.5% (mostly males, 52.5%) never participated in any MDA rounds.^26^ Another cross-sectional study in Yadgir, India, conducted among 315 elderly individuals (>60 years), reported that 16.5% had never participated in MDA previously.^25^ A recent study in three blocks in the Bidar district (n=1227) reported that 41.5% of individuals (mostly males aged above 19 years) were never treated during the two recent IDA-MDA rounds^27^ and reported that filarial infection in terms of filarial antigen and Mf was significantly higher (p<0.001) among those who never participated in any rounds of MDA. Based on the available literature, various reasons for never being treated are summarised in figure 5.

A recent study in Indonesia (n=1915) showed that nearly half (42%) of respondents were never treated during any round of MDA.^39^ Fear of side effects was the major reason cited, and the authors advocated that the community should be reassured with specific instructions to manage adverse events. A study in Samoa (n=4420) reported that the proportion never treated in different communities ranged between 2.6% and 3.8%, with a marginally higher CFA prevalence of 5.8% compared with those who participated (4.9%) in any one of the rounds.^20^ Another study in American Samoa (n=1881), which reported 6% of never treated individuals, showed that participating in at least one MDA was more protective (OR 0.39, 95% CI 0.16 to 0.96) compared with non-participation.^40^ However, two other studies from American Samoa reported higher percentages (31% and 58%) of never treated.^41 42^ It may be noted that none of the above studies were exclusively conducted on the never treated individuals, and therefore less information is available on their sociodemographic features, filarial infection status, and the various factors and barriers influencing their non-participation. Given India’s commitment to achieving LF elimination, filling this knowledge gap is of high programmatic relevance.

Brady *et al* reported that there may be higher proportions of never treated individuals, especially among hard-to-reach populations and among individuals with limited awareness about LF/MDA.^17^ In a few settings, more men were never treated because of their absence due to the nature of their work, while in a few other settings, women were more often never treated due to repeated pregnancies during the MDA rounds.^17^ Modelling studies also have shown that the proportion never treated can strongly influence the achievement of LF elimination^33 43^ and its impact is greater in areas with high transmission.^28 44^

The problem of never being treated was identified as one of the research priority areas during the WHO-RTAG meeting held in Kathmandu, Nepal, in June 2024.^21^ The present protocol was an outcome of this meeting, considering the importance of never-treated individuals, in discussion with the national programme. Identifying and characterising never treated individuals will help the programme to improve MDA delivery strategies and activities to improve access and compliance in the community.

The proposed study integrates both quantitative and qualitative designs to understand the levels of filarial infection among the never treated and identify the various factors influencing their non-participation, such as sociocultural, behavioural and programmatic drivers. This will enable the designing of tailored communication and community engagement strategies to tackle the issue of never treatment. Understanding demand-side (never treated individuals) and supply-side (programme level) challenges is equally important. Workforce shortages, drug supply chain issues, weak supervision and difficulty in reaching remote or mobile populations have frequently been cited as barriers to effective MDA coverage in India.^14 45 46^ The present study will enable us to identify and address various challenges with suitable and feasible interventions as per local needs. For instance, in Indonesia, motivating drug distributors to identify never treated was suggested as a key strategy, while in Myanmar, adjusting the timing of drug distribution and strengthening mop-up activities were recommended.^18 38^

The national programme has taken measures to improve drug compliance in the community, like training of healthcare workers and CDAs on all aspects of MDA, enumeration, management of adverse events and undertaking refusal conversion, if any. The CDAs are motivated to be actively involved by providing incentives for their work. Further, robust social mobilisation through IEC campaigns by influential members of the community and non-governmental organisations has been done to increase community awareness and thereby encourage their active participation in MDA. The programme implements special mop-up rounds to reach out to the missed individuals, migrants, nomadic populations and those in hard-to-reach areas and terrains.^12^

### Strengths and limitations

This is the first study to document filarial infection among the never treated individuals with a large sample size of 10 140 individuals from 4 districts with high endemicity. This sample size has sufficient power to estimate the anticipated CFA and MF prevalence among the never treated and therefore the results can be generalised. Another major strength of this protocol is its mixed-method design, as it integrates both epidemiological and social science approaches to understand various factors influencing their systematic non-participation in MDA.

Although we proposed to enumerate never treated individuals from the MDA registers available in the health facility; however, we understood from the state and district officials that information on never treated individuals sometimes is not recorded or is incomplete in the MDA registers. Therefore, to overcome this gap, the community health workers will be probed in-depth to identify the systematic defaulters of MDA and to supplement, household surveys will be conducted, and the individuals will be enquired for non-participation during MDA till the required sample size is achieved. Although we have restricted the recall up to 5 years to collect information on non-participation in MDA, there could be an element of bias in view of the long duration and, also due to drug administration under other national programmes for prevention of soil transmitted helminths and anaemia. All efforts will be made to minimise the recall bias by eliciting in-depth information from the participants.

### Ethics and dissemination

This study has been approved by the ICMR-VCRC institutional ethics committee (IHEC 07-0824/N/F, dated 25 September 2024). Administrative approvals necessary to conduct the study in the selected districts have been obtained from the State and District NCVBDC offices.

Prior to testing of the study participants, a written informed consent will be obtained from all adults aged≥18 years. For children aged 5–17 years, a written informed consent will be obtained from the parent or legally authorised representative. Additionally, a verbal or written assent, as applicable, will be obtained from all children aged 7–17 years. As for sociological surveys, a written informed consent will be obtained before the participant is interviewed.

Following the completion of the study, a dissemination workshop will be held with stakeholders at different levels: national (policymakers, administrators, finance authorities and programme), state, district and the community (local community groups and others) to highlight the importance of identifying the never treated, the study results and the need for devising site-specific strategies to enhance their participation in MDA. Results will be presented at scientific meetings and published in international peer-reviewed journals. A final summary report will be provided to the funders of the study.

### Patient and public involvement

Patients or the public were not involved in the design, or conduct, or reporting, or dissemination plans of our research.
