# Supplementary material for: Cross-sectional study to assess filarial infection among the never treated individuals in selected districts in India: a study protocol
Source: BMJ Open. 2026 Mar 19;16(3):e113797. doi: 10.1136/bmjopen-2025-113797 (PMC13007103; doi:10.1136/bmjopen-2025-113797)
Supplement: Supplementary file 1 [file bmjopen-2025-113797.reviewer_comments.pdf]

PEER REVIEW HISTORY

BMJ Open publishes all reviews undertaken for accepted manuscripts. Reviewers are asked to complete a checklist review form and are provided with free text boxes to elaborate on their assessment. These free text comments are reproduced below.

ARTICLE DETAILS

Title (Provisional)

A cross-sectional study to assess filarial infection among the never treated individuals in selected districts in India: a study protocol

Authors

Dinesh, Raja J; Srividya, Adinarayanan; M M, Muhammed Jabir; Kishanthini, Manikandan; Dogra, Vishal; Tripathi, Bhupendra; Sharma, Rinku; Jain, Tanu; Rahi, Manju

---

VERSION 1 - REVIEW

**Reviewer**	**1**
**Name**	**de SOUZA, Dziedzom K.**
**Affiliation**	**University of Ghana Noguchi Memorial Institute for Medical Research**
**Date**	**21-Jan-2026**
**COI**	**None**

Overall, the paper is well described. The following comments will help improve the clarity of the paper.

- Title: This should indicate the study design

- Study population: clarify how the MDA registers will be used to identify the never-treated. Describe the MDA registers in India and the type of information these contain, their limitations and how they will enable the identification of the never-treated. There are a couple of studies that looked at MDA registers. It might be useful to refer to these.

- Do ASHAs and ANMs have records of people who are never-treated? How would these people enable the identification of the never-treated? A possible limitation in using these people is the assumption that the same people have distributed the drugs in the last 5 years. If they have not, then their use in identifying the never-treated may be problematic.

- Exclusion criteria: as written it sounds like this is limited to children you are less than 2 years or are seriously ill. Clarify the "seriously ill" to indicate that these are community members who are seriously ill.

- Sample size: Indicate the total sample size for the study if 2535 people are needed per block.

- the section on KAP surveys should be moved the "Sample size for Qualitative surveys"

- Data analysis: Which "variables" are the authors referring to? Are these the outcome indicators in Table 2? Clarify.

- Prevalence estimates should be accompanied by their 95% CI values.

- Include the dates (period) of the study in the protocol.

VERSION 1 - AUTHOR RESPONSE

POINTWISE AUTHORS REPLY TO COMMENTS BY REVIEWER 1

Comment 1: Overall, the paper is well described. The following comments will help improve the clarity of the paper.

Response: The authors thank the reviewer for the constructive comments.

Comment 2: Title should indicate the study design

Response: The title is revised now as follows: 'Never treated individuals in MDA in India: A probable hidden reservoir for transmission impacting elimination of lymphatic filariasis – protocol of a cross-sectional study' (Lines 1-3)

Comment 3: Study population: clarify how the MDA registers will be used to identify the never-treated. Describe the MDA registers in India and the type of information these contain, their limitations and how they will enable the identification of the never-treated. There are a couple of studies that looked at MDA registers. It might be useful to refer to these.

Response: We have described the MDA registers and how they will be used to identify the never treated individuals for better clarity in the revised manuscript as follows:
'ASHAs and ANMs are village level healthcare workers who act as a link between the community and the primary healthcare system in the country, catering to a population of 1000 approximately. They are local residents of the village and are well known to the people in the community as they carry out regular household visits as a part of their responsibilities. They usually maintain a family register containing sociodemographic and other healthcare related details of all inmates in the households in their allotted area. Further, they are also well trained to be drug administrators during the MDA rounds and maintain records in the form of MDA registers (year-wise) which contain sociodemographic details of beneficiaries and defaulters during the MDA rounds'. (Lines 207-222)

In the event of incomplete records in MDA register or non-availability of such registers or ASHAs are unaware of never treated households, the study team will systematically sample HHs and probe the inmates for non-participation in MDA, till the required sample size for that village is achieved. Information will be supplemented with the data from other sources like WHO monitoring teams, non-governmental organizations and coverage evaluation surveys, if available. (Lines 222-230)

Comment 4: Do ASHAs and ANMs have records of people who are never-treated? How would these people enable the identification of the never-treated? A possible limitation in using these people is the assumption that the same people have distributed the drugs in the last 5 years. If they have not, then their use in identifying the never-treated may be problematic.

Response: ASHAs and ANMs are village level healthcare workers who act as a link between the community and the primary healthcare system in the country, catering to a population of 1000 approximately. They are local residents of the village and are well known to the people in the community as they carry out regular household visits as a part of their responsibilities. They usually maintain a family register containing sociodemographic and other healthcare related details of all inmates in the households in their allotted area. Further, they are also well trained to be drug administrators during the MDA rounds and maintain records in the form of MDA registers (year-wise) which contain sociodemographic details of beneficiaries and defaulters during the MDA rounds. This is now clearly mentioned in the revised manuscript for better clarity (Lines 207-222)

Comment 5: Exclusion criteria: as written it sounds like this is limited to children you are less than 2 years or are seriously ill. Clarify the "seriously ill" to indicate that these are community members who are seriously ill.

Response: Accepted. The sentence is modified now for better clarity in the revised manuscript as: 'Children aged less than 2 years, and those individuals found to be seriously ill or bed-ridden at the time of survey will be excluded' (Lines 167-168)

Comment 6: Sample size: Indicate the total sample size for the study if 2535 people are needed per block.

Response: The suggestion is accepted and the sample size is now mentioned clearly in the revised manuscript as follows: 'a total of 10,140 never treated individuals will be screened for filarial infection from four blocks' (Lines 173-174)

Comment 7: the section on KAP surveys should be moved the "Sample size for Qualitative surveys"

Response: Accepted. The section on KAP surveys is moved to the "Sample size for Qualitative surveys" in the revised manuscript (Lines 176-180)

Comment 8: Data analysis: Which "variables" are the authors referring to? Are these the outcome indicators in Table 2? Clarify.

Response: Agreed. The sentence is now modified as follows for better clarity: 'Univariate analysis will be performed to find the association of infection status (Mf/CFA positivity) with various variables such as age, gender, education, occupation, socioeconomic status, and duration of residency'. (Lines 344-347)

Comment 9: Prevalence estimates should be accompanied by their 95% CI values.

Response: Yes, the prevalence estimates will be provided with 95% CI values (Line 344)

Comment 10: Include the dates (period) of the study in the protocol.

Response: Accepted and included in the newly added sub-section “Study status" under Methods section (Lines 357-360)
